# Detection of human papillomavirus in dental biofilm and the uterine cervix of a pregnant adolescent: Detecção do papilomavírus humano em biofilme dental e no colo uterino de uma adolescente grávida

**DOI:** 10.1590/1516-3180.2014.8812810

**Published:** 2015-04-14

**Authors:** Édila Figuerêdo Feitosa Cavalcanti, Célia Regina Silva, Dennis Carvalho Ferreira, Mariana Vasconcellos Martins Ferreira, Patrícia Rosa Vanderborght, Maria Cynésia Medeiros Barros Torres, Sandra Regina Torres

**Affiliations:** I MSc. Postgraduate Student, Department of Oral Pathology and Diagnosis, School of Dentistry, Universidade Federal do Rio de Janeiro (UFRJ), Rio de Janeiro, Brazil.; II MD, MSc. Associate Professor, Department of Gynecology, School of Medicine, Universidade Federal do Rio de Janeiro (UFRJ), Rio de Janeiro, Brazil.; III PhD, Postgraduate Dentistry Program, Discipline of Oral Medicine, School of Dentistry, Veiga de Almeida University, Rio de Janeiro, Brazil.; IV Postgraduate Student, School of Dentistry, Universidade Federal do Rio de Janeiro (UFRJ), Rio de Janeiro, Brazil.; V PhD. Quality Manager, Department of Clinical Research, D’Or Institute for Research and Education (IDOR), Rio de Janeiro, Brazil.; VI PhD. Associate Professor, Department of Dental Clinic, School of Dentistry, Universidade Federal do Rio de Janeiro (UFRJ), Rio de Janeiro, Brazil.; VII PhD. Associate Professor, Department of Oral Pathology and Diagnosis, School of Dentistry, Universidade Federal do Rio de Janeiro (UFRJ), Rio de Janeiro, Brazil.

**Keywords:** Human papillomavirus 16, Adolescent, Dental plaque, Gingivitis, Periodontitis, Pregnancy

## Abstract

**CONTEXT:**

Adolescence and pregnancy are considered to be risk factors for human papillomavirus (HPV) infection. The relationship between this infection in the uterine cervix and oral HPV infection is controversial.

**CASE REPORT:**

This report describes a case of a pregnant 16-year-old adolescent who presented HPV infection in the uterine cervix and the mouth. Smears were collected from the cervix and the tongue/palate. Dental biofilm samples were also collected. The microarray technique was used to detect HPV. The HPV 56 subtype was observed in the cervical smear and HPV 6 in dental biofilm.

**CONCLUSION:**

In this pregnant adolescent, HPV infection was present in both the cervix and the mouth, but the HPV subtypes infecting these two areas were different.

## RESUMO

**CONTEXTO:**

A adolescência e a gestação são consideradas fatores de risco para a infecção pelo papilomavírus humano (HPV). A relação entre essa infecção no colo do útero e na boca num mesmo paciente é controversa.

**RELATO DE CASO:**

Descrever o caso de uma adolescente grávida de 16 anos que apresentou a infecção pelo HPV no colo do útero e na boca. Esfregaços foram realizados no colo do útero e em língua/palato. Amostras de biofilme dental também foram coletadas. Para detectar o HPV, foi utilizada a técnica do microarranjo. O HPV 56 foi o subtipo encontrado no esfregaço cervical e o tipo HPV 6 no biofilme dental.

**CONCLUSÕES:**

Observamos, nessa adolescente grávida, a presença do HPV na boca e no colo do útero, mas os subtipos virais que infectavam essas duas regiões eram distintos.

## INTRODUCTION

Human papillomavirus (HPV) infection is the most common sexually transmitted disease (STD). The reported risk factors for HPV infection are multiple partners, oral contraceptive and an early start to sexual activity.^1^ The hormonal alterations that occur during pregnancy and puberty may predispose towards immunosuppression and result in persistence of HPV infection and progression of lesions.^2^^,^^3^

In order to promote the health of both mother and child, pregnant women need to adhere to primary healthcare measures. Cervical screening and health education strategies are powerful tools for gaining knowledge about HPV. The treatment for the induced lesions ranges from home-based therapy to surgical therapy. The present case report describes a 16-year-old patient who was diagnosed with cervical and oral HPV infection.

## CASE REPORT

A 16-year-old pregnant adolescent attended a routine prenatal consultation at the Maternity School of the Federal University of Rio de Janeiro (Universidade Federal do Rio de Janeiro, UFRJ). According to her medical records, she had a history of syphilis and bacterial vaginosis that had been adequately treated at the same health clinic. In the clinical examination performed during the first trimester of pregnancy, she presented cervical lesions diagnosed as HPV-induced lesions in the cytopathological evaluation. The patient received guidance regarding her HPV infection and for periodic monitoring of the cervical lesions. Moreover, aspects of STD prevention, including oral sex, and family planning issues were addressed.

At the time of the clinical appointment, she was 31 weeks pregnant with her first child. She had been using an oral contraceptive method, which failed due to incorrect use, and resulted in an unwanted pregnancy. When interviewed, she reported that her active sex life started when she was 13 years old and that she had had 10 sexual partners since her first sexual intercourse, but that at that moment, she had only one sexual partner. During the interview, she also reported having vaginal intercourse and oral sex at weekly frequency. The last vaginal and oral intercourse had been on the previous day and four days earlier, respectively. She stated that she had never used alcohol, marijuana or cocaine.

In the genital examination, white stained areas were identified in the uterine cervix, after applying acetic acid. Local smears for cytopathological analysis were collected from these areas. The slides were sent to the laboratory at the same clinic. An oral examination was performed, including a complete periodontal examination. The patient did not present any oral lesions and her teeth were healthy. The gingival bleeding index, dental plaque index and the mean probing depth of the gingival sulcus were recorded. The patient presented bleeding in 9% of the gingival sites and dental biofilm in 65% of the tooth sites. The mean probing depth was 2.24 mm, with no attachment loss.

**Figure 1.**
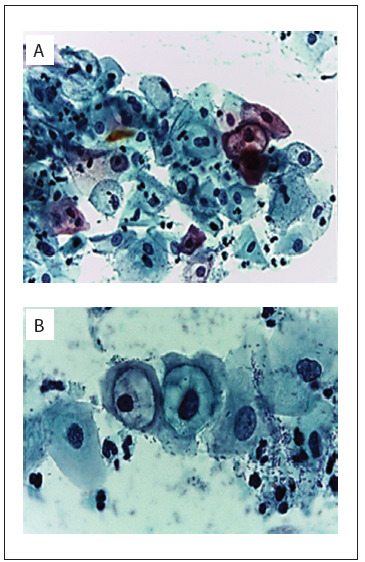
Micrograph showing a low-grade squamous intraepithelial lesion (LSIL, CIN1): (A) cells exhibiting vacuoles, atypical mitosis and different sizes of nucleus, with Pap staining, 400 X; (B) koilocytosis halo image, with Pap staining, 1,000 X.

The slides sent to the laboratory were processed using the Papanicolaou (Pap) test. The cytological data were evaluated by means of the Bethesda criteria and the cells were found to present koilocytosis, nuclear enlargement, nuclear hyperchromasia, irregular nuclear outlines and perinuclear halos (Figure 1).

Swab samples for HPV identification were collected from the cervix and the mouth (dorsum of the tongue and the area of the soft and hard palate). Samples of dental biofilm were also collected using a sterile periodontal curette, from the teeth with deeper sulci. The samples collected were subjected to DNA purification (QIAamp DNA Mini Kit, Qiagen) and the microarray reaction for DNA HPV genotyping (Papillocheck, Greiner-Bio-One).

The cervical smear showed the presence of DNA from HPV56, which is a high-risk subtype. No HPV DNA was detected in the tongue/palate smear. DNA from HPV 6 was detected in one of the four samples of dental biofilm. HPV was present in the periodontal sulcus of the lower left canine. The depth of the gingival sulcus at this site was 4 mm, and it did not present bleeding on probing.

Preventive measures were reaffirmed and a clinical monitoring approach was implemented in relation to the cervix HPV-induced lesions, which consisted of cervical screening every 12 months. The lesions presented a self-healing course within the first year of observation.

## DISCUSSION

The presence of HPV-infected lesions is sometimes only shown through screening techniques.^4^^,^^5^ In this case report, a patient presented asymptomatic cervical lesions that were suggestive of HPV infection, which were identified through white staining after acetic acid application, and were diagnosed by means of cytopathological examination as low-grade intraepithelial lesions. Identification of high-risk HPV56 in the cervical lesions showed the importance of using molecular techniques for identifying the subtype of the virus.

The HPV56 viral subtype detected in this pregnant adolescent is among the high-risk oncogenic subtypes that might be found in adolescents.^6^ It is also frequently found in populations of adult women in countries such as Kuwait, Germany, Philippines, United States and Brazil.^7^

The protocol for monitoring low-grade squamous intraepithelial lesions in adolescents (≤ 20 years) consists of performing cytological tests every 12 months, for a period of two years.^6^ These lesions do not require treatment, because of their transient and self-healing course.^6^ Thus, the clinical monitoring approach was used for the patient in this case report. In fact, the lesions in this patient self-healed during the first year of the follow up.

Presence of HPV DNA has been observed under some pathological conditions that affect the mouth. This virus has been identified in abscesses of endodontic origin; in gingivitis and periodontitis; and in malignant tumors, such as oral squamous cell carcinoma.^8^ HPV may play a role in facilitating periodontal disease.^9^ Periodontitis is an inflammatory disease induced by specific infectious bacteria, which causes destruction of the supporting dental tissues with consequent tooth loss. The clinical characteristics of periodontitis include tissue inflammation, which results in loss of clinical attachment and alveolar bone and development of periodontal sulci. Chronic periodontitis is the most common type of periodontal disease, and it is more prevalent in adults; however, it may affect individuals at any age.

Some human viruses have been implicated in facilitating establishment of periodontal pathogenic bacteria in the gingival sulcus. HPV may infect deeper layers and reach the basal cell layer without necessarily causing any type of lesion, because the junctional epithelium connects the gingival sulcus with the connective tissue through a gap in the epithelial barrier.^10^ Therefore, gingival tissues could act as a reservoir for the virus and release pro-inflammatory cytokines, thereby causing instability of the cell defense mechanisms and contributing towards development of periodontitis.

Even though the patient reported a recent history of oral sex, she did not present HPV in the cells collected from the mouth smears. The oral site from which HPV DNA was observed in this pregnant adolescent was in dental biofilm from a periodontal sulcus of an anterior tooth that did not present any clinical signs of inflammation or deep sulci. Detection of HPV at this site may suggest that the anterior sites of the mouth were exposed to HPV during oral intercourse. Other authors did not believe that periodontal tissues were able to act as a reservoir for HPV, because they were unable to observe the virus in any sample of gingivitis, periodontitis or healthy gingival tissue in their study.^9^

Little is known about the prevalence of this infection in the mouth. The practice of oral sex is currently suggested to be a risk factor for HPV infection in the mouth.^1^^,^^11^ Other forms of HPV transmission have been described, such as kissing, contact through hands due to sexual practices like masturbation, maternal-fetal transmission and, rarely, by means of self-inoculation and fomites.^5^

Some studies have attempted to find evidence of an association and/or relationship between HPV infection in the mouth and genitalia of pregnant women.^4^^,^^12^ The studies that have reported HPV infection in the mouth and genitalia in pregnant women are listed in Table 1. These studies observed presence of HPV at both of these anatomical sites. The mouth is a region of many anatomical peculiarities that comprises both hard and soft tissues. The latter has both keratinized and non-keratinized components. These features may prevent the HPV infection in the mouth. However, they found no evidence of any association between subtypes.^4^^,^^12^

**Table 1.**
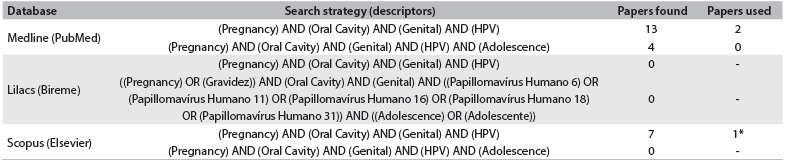
Database search results for the relationship between HPV infections in the mouth and genitalia among pregnant women. Search performed on April 8, 2014

*One of two studies also found in PubMed database.

## CONCLUSIONS

In this pregnant adolescent, there were different subtypes of HPV infection affecting the uterine cervix and the mouth. Further clinical studies with data on individual characteristics and behavior are needed, in order to determine the real risk factors for HPV infection in the mouth.

## References

[1] D’Souza G, Agrawal Y, Halpern J, Bodison S, Gillison ML (2009). Oral sexual behaviors associated with prevalent oral human papillomavirus infection. J Infect Dis.

[2] Smith EM, Johnson SR, Jiang D (1991). The association between pregnancy and human papilloma virus prevalence. Cancer Detect Prev.

[3] Insinga RP, Dasbach EJ, Elbasha EH (2009). Epidemiologic natural history and clinical management of Human Papillomavirus (HPV) Disease: a critical and systematic review of the literature in the development of an HPV dynamic transmission model. BMC Infect Dis.

[4] Smith EM, Ritchie JM, Yankowitz J (2004). HPV prevalence and concordance in the cervix and oral cavity of pregnant women. Infect Dis Obstet Gynecol.

[5] Dos Reis HL, Rabelo PC, de Santana MR, Ferreira DC, Filho AC (2009). Oral squamous papilloma and condyloma acuminatum as manifestations of buccal-genital infection by human papillomavirus. Indian J Sex Transm Dis.

[6] Brown DR, Shew ML, Qadadri B (2005). A longitudinal study of genital human papillomavirus infection in a cohort of closely followed adolescent women. J Infect Dis.

[7] Al-Awadhi A, Chehadeh W, Al-Jassar W (2013). Phylogenetic analysis of partial L1 gene of 10 human papillomavirus types isolated most commonly from women with normal and abnormal cervical cytology in Kuwait. Arch Virol.

[8] Hormia M, Willberg J, Ruokonen H, Syrjänen S (2005). Marginal periodontium as a potential reservoir of human papillomavirus in oral mucosa. J Periodontol.

[9] Parra B, Slots J (1996). Detection of human viruses in periodontal pockets using polymerase chain reaction. Oral Microbiol Immunol.

[10] Schroeder HE, Listgarten MA (1997). The gingival tissues: the architecture of periodontal protection. Periodontol 2000.

[11] Brondani M (2008). HPV, oral sex, and the risk of oral cancer: food for thought. Spec Care Dentist.

[12] Rintala M, Grénman S, Puranen M, Syrjänen S (2006). Natural history of oral papillomavirus infections in spouses: a prospective Finnish HPV Family Study. J Clin Virol.

